# The prognosis biomarkers based on m6A-related lncRNAs for myeloid leukemia patients

**DOI:** 10.1186/s12935-021-02428-3

**Published:** 2022-01-07

**Authors:** Li-Rong Yang, Zhu-Ying Lin, Qing-Gang Hao, Tian-Tian Li, Yun Zhu, Zhao-Wei Teng, Jun Zhang

**Affiliations:** 1 Department of Oncology, The Third People’s Hospital of Chengdu, The Affiliated Hospital of Southwest Jiaotong University, 82 Qinglong Road, Chengdu, 610031 Sichuan China; 2 Kunming Medical University, Kunming, 650000 Yunnan China; 3 Yunnan Key Laboratory of Digital Orthopedics, Department of Orthopedic, The First People’s Hospital of Yunnan Province, Kunming, 650000 Yunnan China; 4 The Sixth Affiliated Hospital of Kunming Medical University, The People’s Hospital of Yuxi City, Yunnan, 653100 Yuxi China; 5 Center for Life Sciences, School of Life Sciences, Yunnan University, Kunming, 650000 China

**Keywords:** m6A, m6A-related lncRNAs, CRNDE, CHROMR, NARF-IT1, Myeloid leukemia, Prognosis

## Abstract

**Background:**

Chronic myeloid leukemia (CML) and acute myeloid leukemia (AML) are two common malignant disorders in leukemia. Although potent drugs are emerging, CML and AML may still relapse after the drug treatment is stopped. N6-methyladenosine (m6A) and lncRNAs play certain roles in the occurrence and development of tumors, but m6A-modified LncRNAs in ML remain to be further investigated.

**Methods:**

In this study, we extracted and analyzed the TCGA gene expression profile of 151 ML patients and the clinical data. On this basis, we then evaluated the immune infiltration capacity of ML and LASSO-penalized Cox analysis was applied to construct the prognostic model based on m6A related lncRNAs to verify the prognostic risk in clinical features of ML. Quantitative reverse transcription PCR was used to detect the expression level of LncRNA in in ML cell lines K562, MOLM13 and acute monocytic leukemia cell line THP-1.

**Results:**

We found 70 m6A-related lncRNAs that were related to prognosis, and speculated that the content of stromal cells and immune cells would correlate with the survival of patients with ML. Next, Prognostic risk model of m6A-related lncRNAs was validated to have excellent consistency in clinical features of ML. Finally, we verified the expression levels of CRNDE, CHROMR and NARF-IT1 in ML cell lines K562, MOLM13 and acute monocytic leukemia cell line THP-1, which were significant.

**Conclusions:**

The research provides clues for the prognosis prediction of ML patients by using the m6A-related lncRNAs model we have created, and clarifies the accuracy and authenticity of it.

**Supplementary Information:**

The online version contains supplementary material available at 10.1186/s12935-021-02428-3.

## Background

Chronic myeloid leukemia (CML) and acute myeloid leukemia(AML) are two common malignant disorders in leukemia [1]. They have different forms and different age distributions, AML mostly occur in older adults, the significance is increasing in patients aged ≥ 60 years and the incidence is higher in males than in females [2]. CML is very rare in early childhood, but once it happens in children and adolescents, it tends to present with more aggressive characteristics [3, 4]. With the discovery of signal pathways and molecular biological markers, there are increasing number of researchers have devoted to the study of the mechanism of myeloid leukemia.

LncRNAs are defined as a group of non-coding transcripts more than 200 nt in length, that are unable to encode protein [5]. LncRNAs are involved in almost all the disease processes, such as diabetes, heart diseases, and different types of cancers. And they have been involved in various mechanisms, for instance, regulation of transcription, protein modification, and translation [6]. Recently, more attention has been paid to its modulation functions, especially in cancer initiation, progression and metastasis [7, 8]. However, N6-methyladenosine (m6A)-related LncRNAs have been rarely studied in myeloid leukemia.

m6A is the most prevalent form of RNA modifications, it is involved in cellular processes, such as cell cycle regulation, differentiation, and invasion. And it regulates mRNA expression [9]. A large amount of evidence indicates that both m6A and lncRNA play a certain role in the occurrence and development of tumors [10, 11]. In addition, m6A is essential for maintaining the differentiation process in the hematopoietic system. For example, m6A-forming enzyme METTL3 promotes the expression of BCL2, c-MYC and PTEN in human AML MOLM13 cells to control myeloid differentiation [12], and changes in the m6A regulatory gene lead to poor survival in AML patients [13]. Despite the advances have been made, m6A-modified LncRNAs in the regulation of ML remain to be further investigated.

In our study, we extracted the TCGA gene expression profile of 151 ML patients and the corresponding clinical data and analyzed 70 m6A-related lncRNAs that were related to survival and prognosis. On this basis, we evaluated the percentage and difference of each kind of immune cell and analyzed the ImmuneScore, the StromalScore and ESTIMATEScore of all ML patients. Next, we used the LASSO-penalized Cox analysis method to construct a prognostic model based on m6A related lncRNAs to verify the prognostic risk in clinical features of ML. Finally, we verified the expression levels of m6A-related lncRNAs in ML cell lines K562, THP1, and MOLM13.

## Materials and methods

### Data of patients with ML

The gene expression profiles of 151 patients with ML and corresponding patients’ clinical profiles dataset including outcome, age and survival time were downloaded from the TCGA database (https://gdc.nci.nih.gov/) [14]. The samples origin used for studying the gene expression of lncRNA from the TCGA database were bone marrow. The specific raw data information were in Additional file 1: Table S1.

### Selection of m6A-related lncRNAs

We obtained the LncRNA related to m6A genes from the gene expression file. Related data was analyzed by the limma and igraph package in R version 4.1.0. The process used the criteria of correlation coefficient > 0.5 and *p* < 0.001, and 525 m6A-related lncRNAs were identified. According to previous studies, the 23 m6A genes include 8 writers (METTL3, METTL14, METTL16, WTAP, VIRMA, ZC3H13, RBM15 and RBM15B), 13 readers (YTHDC1, YTHDC2, YTHDF1, YTHDF2, YTHDF3, HNRNPC, FMR1, LRPPRC, HNRNPA2B1, IGF2BP1, IGF2BP2, IGF2BP3, and RBMX), and 2 erasers (ALKBH5 and FTO) [11]. Then we analyzed m6A-related lncRNAs related to survival with survival R package, samples were screened according to *p*-value < 0.01.

### Differentially Expressed Genes (DEGs) analysis

The consensus clustering analysis of 140 TCGA ML tumor samples was performed by using the ConsensusClusterPlus R package (parameters: reps = 50, pItem = 0.8, pFeature = 1, distance = “euclidean”). ConsensusClusterPlus is a class discovery tool with confidence evaluation and project tracking [15]. Furthermore, DEGs were analyzed and visualized by limma R package and ggplot2 R package between cluster1 and cluster2, male and female, or age > 60 and ≦ 60 in ML. Heatmap of DEGs was drawn using pheatmap R package. The screening criteria were |log2 (fold change) | ≥ 1 and *p*-value < 0.05.

### Immune cells infiltration analysis

The ESTIMATE R package was used to assess overall immune infiltration (ImmuneScore), stromal content (StromalScore) and the combined score (ESTIMATEScore) of cluster1 and cluster2 in ML tumor sample using the downloaded database [16]. To estimate the relative abundance of 22 kinds of tumor-infiltrating immune cells in the ML tumor sample from TCGA, the CIBERSORT was applied to calculate putative proportion of immune cell fraction of cluster1 and cluster2 in ML tumor sample [17]. Related data was analyzed by the vioplot and lima R package. The ggplot2 and ggpubr R package were used to evaluate expression of immune molecules of cluster1 and cluster2. In order to reveal the relationship between immune molecules and m6A-related lncRNAs, we used corrplot R package to assess the regulatory relationship between immune molecules and prognostic-related m6A-related lncRNAs. Samples were screened according to *p*-value < 0.05.

### Establishment and validation of the risk signature

The m6A-related lncRNAs related to survival and prognosis were randomized into the training set and the testing set, the tumor samples of training set and the testing set were divided into high risk group and low risk group according to the median of risk score. The risk score was calculated following formula: Risk score = coef (lncRNA1)◊expr (lncRNA1) + coef (lncRNA2) ◊expr (lncRNA2) + … + coef (lncRNAn) ◊expr (lncRNAn), where coef (lncRNA) represented the coefficient of lncRNAs correlated with survival, and expr (lncRNA) represented the expression of lncRNAs. The least absolute shrinkage and selection operator (LASSO) [18] for Cox regression analyses were used to construct a prognostic model. And we used multivariate and univariate Cox regression analyses to test whether the prognostic model was an independent variable, independent of other clinical characteristics (age, gender) in the patients with ML. The glmnet and caret R package were conducted LASSO Cox regression.

### Establishment of the predicted risk nomogram

The probability of predictors (age, gender) for survival was predicted. And the comparison of a risk score between other predictors (age, gender, ImmuneScore and cluster) was performed. Furthermore, the positive or negative correlations between the risk score and each kind of immune cell was verified.

### Cell line culture

Human CML cell line K562, AML cell line MOLM13 and acute monocytic leukemia cell line THP-1 were maintained in RPMI 1640 medium (Gibco, Grand Island, NY, USA), supplemented with 10% heat-inactivated fetal bovine serum (FBS, Biological Industries, Beit Haemek, Israel), and 100 U/ml penicillin and 10 U/ml streptomycin. And human mesenchymal cell line HS-5 was cultured in DMEM medium (Gibco, Grand Island, NY, USA), supplemented with 15% heat-inactivated FBS. All the cell lines were cultured under 37 °C, 5% CO2, 95% humidified atmosphere. MOLM13 and HS-5 cells were purchased from Cellcook Biotech Co.,Ltd. The other cell lines were obtained from ATCC, USA.

### Quantitative real-time (qRT-PCR) analysis

Total RNA was extracted from cell using the Trizol reagent extraction method. Synthesis of cDNA was operated using the RevertAid First Strand cDNA Synthesis Kit (Thermo Scientific, USA), and qPCR reactions were performed using SsoFastTM EvaGreen Supermix (Bio-Rad, USA) according to the manufacturer’s instructions and reported by the 2^−ΔΔCT^ method. Primers for detection of RNA expression were described in Additional file 2: Table S2.

### Statistical analysis

All data were compared with unpaired t-test. The overall survival (OS) was represented by a Kaplan-Meier curve using survival R package. *p* < 0.05 was considered to be statistically significant. All statistical data were analyzed using Graphpad Prism 6 software (GraphPad Software Inc., San Diego, CA).

## Results

### Identification and consensus clustering analysis of m6A-related lncRNAs in patients with ML

**Fig. 1:**
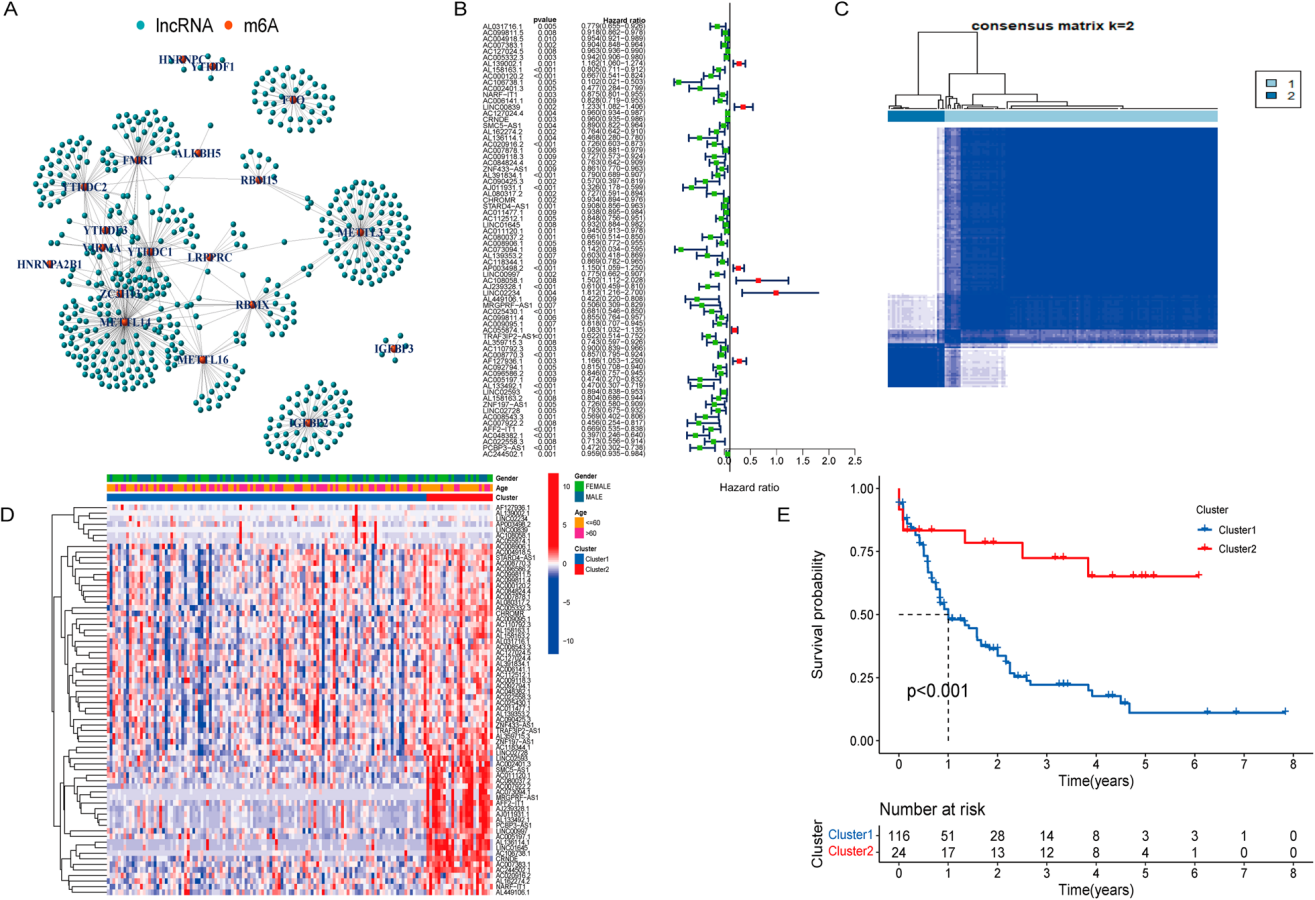
Identification and Consensus Clustering analysis of m6A-related lncRNAs in ML patients. **A** Interaction network diagram for relationship between m6A and LncRNA. **B** Forest diagram showed that 70 m6A-related lncRNAs were correlated to survival. **C** Consensus matrix for k = 2. **D** Heatmap of the clinicopathological features (age, gender) and cluster. **E** Kaplan-Meier overall survival (OS) curves for patients in cluster1 and cluster2. **p < 0.01 and ***p < 0.001

The expression data of 14,086 lncRNAs and 23 m6A genes were obtained from the TCGA database and analyzed. Consequently, the results showed that 19 m6A genes and 525 LncRNAs were correlated (*p* < 0.001), and the relationship between m6A and LncRNA was visualized using interaction network diagram (Fig. 1 A). Therefore, the 525 LncRNAs were named as m6A-related LncRNAs. Then we analyzed the relationship between m6A-associated LncRNAs and survival, and we demonstrated that 70 m6A-related lncRNAs were related to survival and prognosis in 140 tumor samples. These results were displayed using forest diagram (*p* < 0.01) (Fig. 1B).

Besides, we selected the value of k = 2 to reduce the interference between subgroups for consensus clustering analysis (Fig. 1C). The two subgroups of samples were classified into cluster1 (n = 116) and cluster2 (n = 24) (Additional file 1: Table S1). Moreover, we further compared the clinicopathological features (age, gender) between cluster1 and cluster 2 (Fig. 1D). There was no significant distinction between the two subgroups and it indicated that the two clusters were closely related to ML. The overall survival (OS) of cluster 2 was found to be longer than that of the cluster1 (Fig. 1E).

## Immune cells infiltration of two clusters in ML

**Fig. 2:**
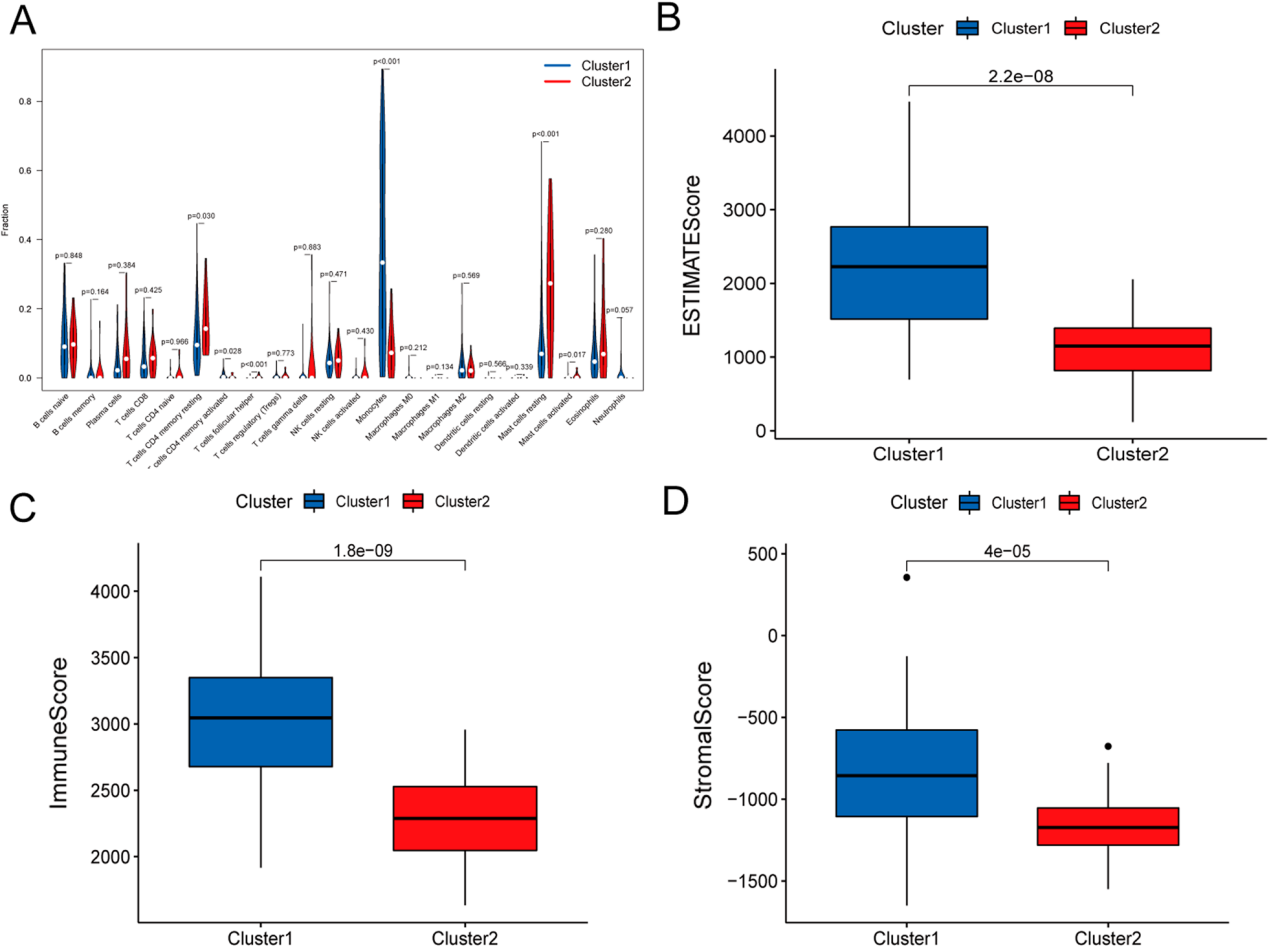
Immune Cells Infiltration of cluster1 and cluster1 in ML. **A** The fraction of 22 immune cell type signature in cluster1 and cluster2 (Wilcox Test). **B**–**D** The comparsion of ESTIMATEScore, ImmuneScore, and StromalScore in cluster 1 and cluster 2 (Wilcox Test). **p < 0.01 and ***p < 0.001

Tumor-infiltrating immune cells were the main component of important prognostic information for ML patients. In order to evaluate the percentage and difference of each kind of immune cells in the 140 tumor samples, CIBERSORT algorithm was used to estimate the fraction of 22 immune cell types between cluster1 and cluster2. Compared to cluster2, we summarized that T cells CD4 memory resting, T cells follicular helper, Mast cells resting and Mast cells activated cover a small proportion in cluster1 (*p* <0.05), while T cells CD4 memory activated and Monocytes accounts for a larger proportion (*p* <0.05) (Fig. 2A). ESTIMATE algorithm was used to analyze the ImmuneScore, the StromalScore and ESTIMATEScore of all patients. This data indicated that the ImmuneScore, StromalScore, and ESTIMATEScore were significantly higher in cluster1. The results showed that both immune scores and stromal scores were meaningful and significantly different in the correlation of subtype classification (Fig. 2B–D).

**Fig. 3:**
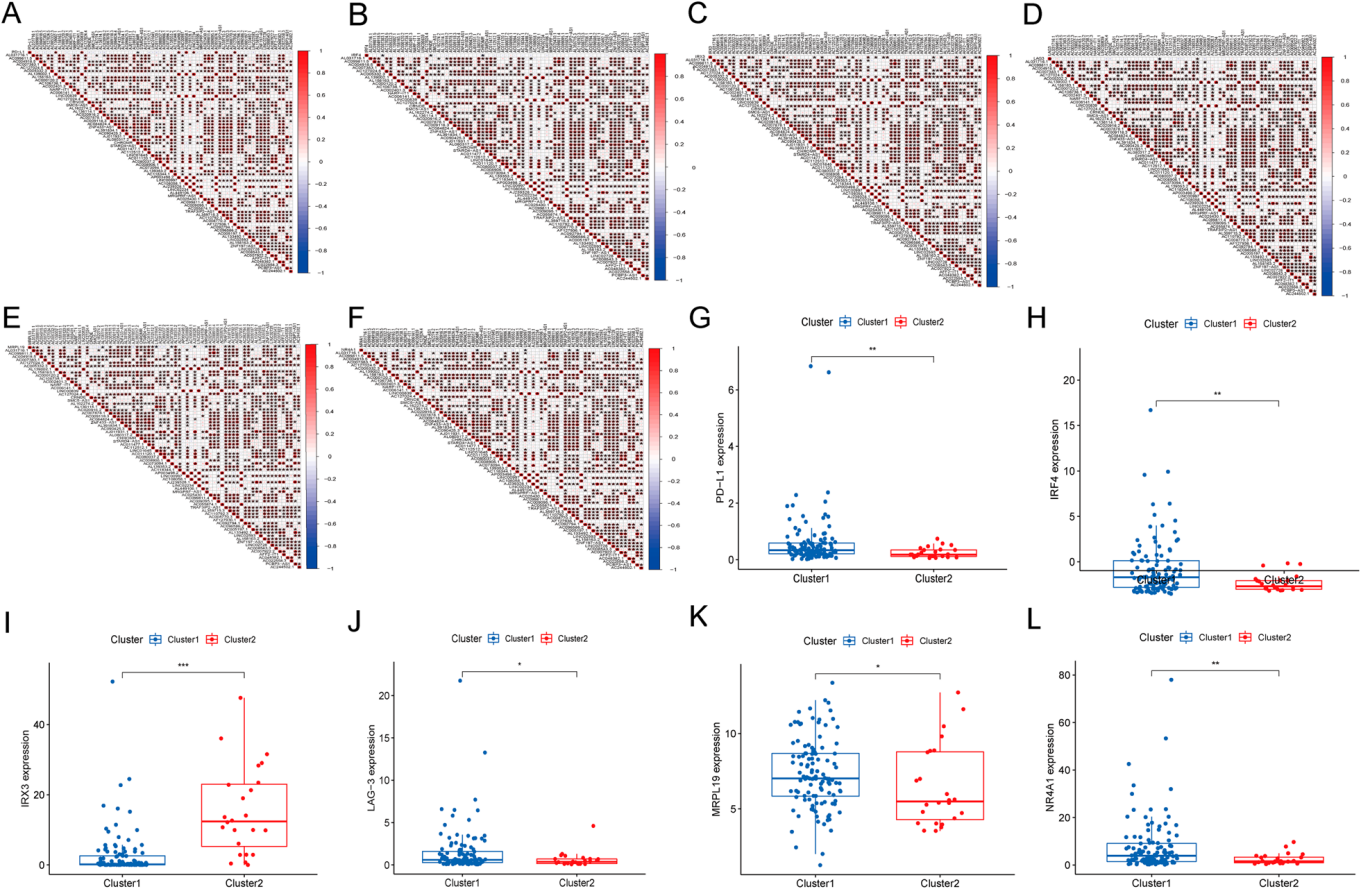
Immune gene analysis in ML. **A**–**F** Interrelationships and correlations between the immune gene (PD-L1, IRF4, IRX3, LAG-3, Tim-3 and NR4A1) and the m6A-associated prognostic lncRNAs. **G**–**L** Immune gene expression of cluster1 and cluster1 in ML, **p < 0.01 and ***p < 0.001

In addition, we analyzed the interrelationships and correlations between the immune genes (PD-L1, IRF4, IRX3, LAG-3, Tim-3 and NR4A1) and the m6A-associated prognostic lncRNAs. There was a strong negative correlation (blue *) and positive correlation (red *) among the immune genes and the m6A-related LncRNAs, results showed in Fig. 3A–F (*p* < 0.05). Almost all the correlations were statistical significant. What’s more, we also substantiated that there was a striking difference in immune gene expression between cluster1 and cluster2 (Fig. 3G–L).

### Construction of prognostic model based on m6A related lncRNAs in ML patients

**Fig. 4:**
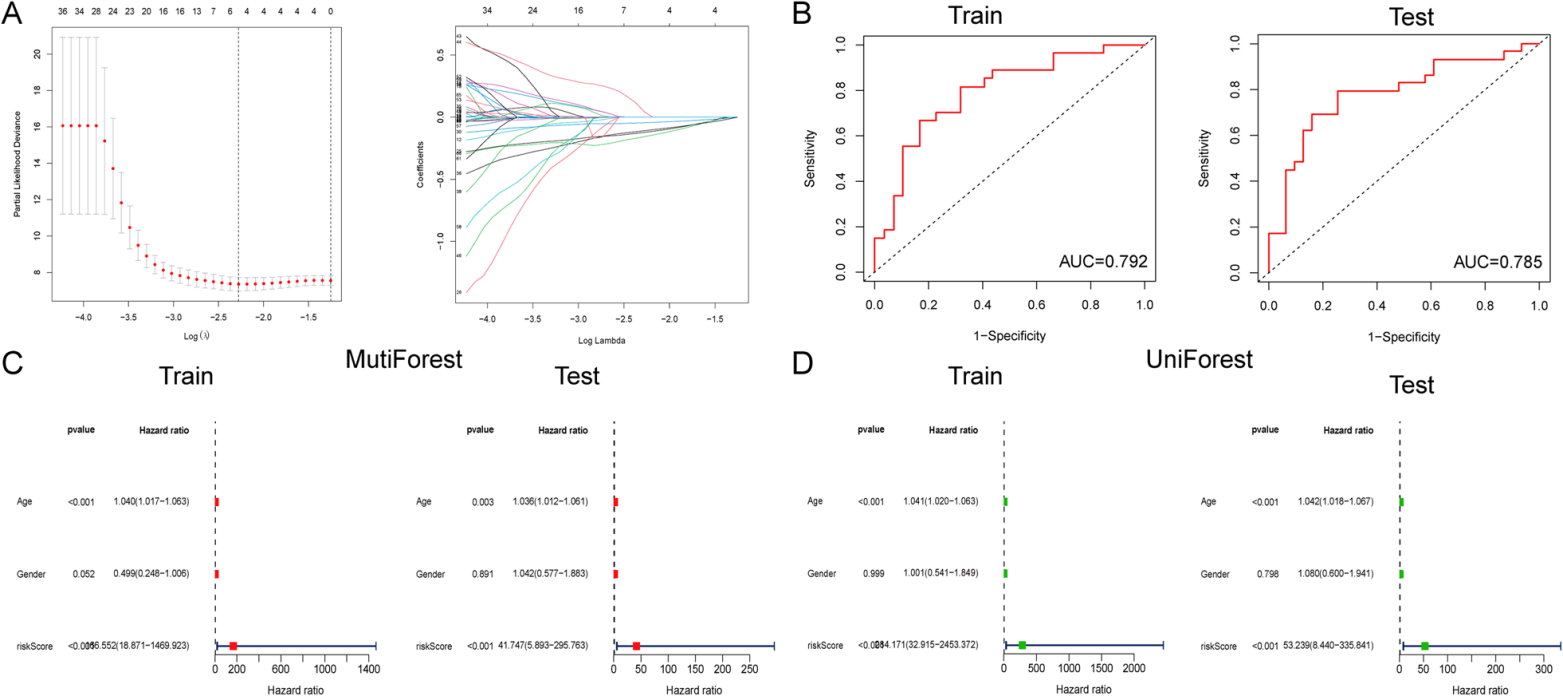
Establishment of prognostic model based on m6A related lncRNAs in ML patients. (A) LASSO Cox regression was conducted to construct the prognostic model. (B) The ROC curves of the risk signature in training and testing group. (C, D) Multivariate and univariate Cox regression analyses of the associated between clinicopathological features (including risk score) and overall survival of patients in the training and testing group. **p < 0.01 and ***p < 0.001

LASSO-penalized Cox analysis is a method to narrow down the scope of gene screening [18], and it is a biased estimation for processing high-dimensional data with an inferior correlation to effectively produce a prognostic indicator. Hence, a prognostic model based on m6A related lncRNAs in ML patients was established using LASSO-penalized Cox analysis, and cross-validation method was used to optimize the prognostic model (Fig. 4A). First, we categorized 140 samples into training group (n = 72) and testing group (n = 68) for multivariate analysis (Additional file 3: Table S3). Next, 140 ML samples were divided into low- and high-risk groups according to the median value of the prognostic risk grade. And we also completed the area under the time-dependent ROC curves (AUCs) analysis to assess the sensitivity and specificity of the prediction of prognostic model. The AUC for 1 year overall survival (OS) was 0.792 for training group and 0.785for testing group (Fig. 4B). Univariate and multivariate Cox regression analysis was used to assess whether the predictive performance and accuracy of our prognostic model can be used as an independent prognostic factor that was independent of other clinical characteristics. The results emphasized that the age (*p* < 0.05) and the risk score (*p* < 0.001 were independent prognostic factors for OS in both testing group and training group (Fig. 4C, D). All these data revealed a good predictive value and high accuracy of this prognostic model based on m6A related LncRNAs.

**Fig. 5:**
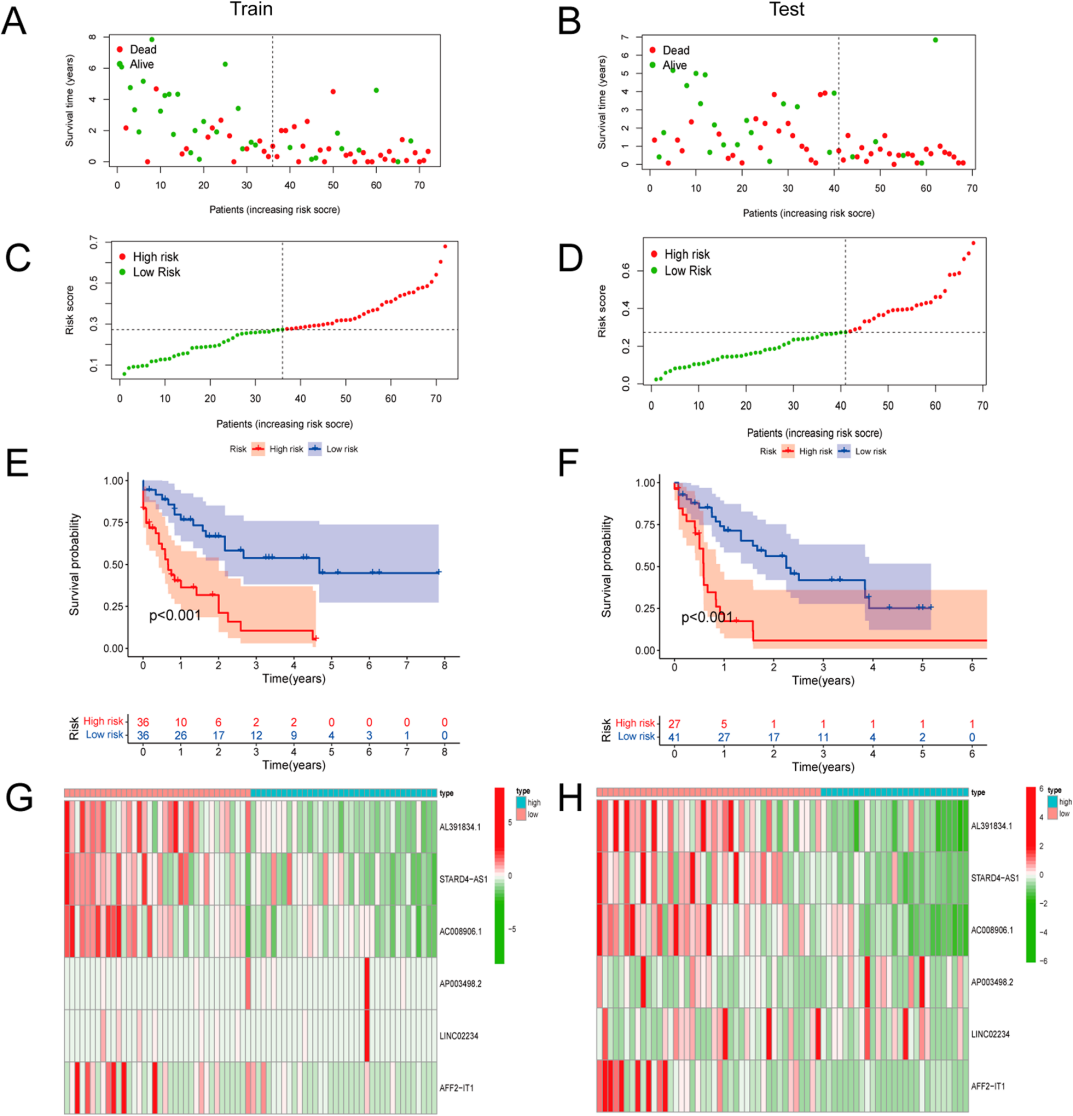
Prognostic value of the risk patterns of the m6A-related lncRNAs in the training and testing set. **A**, **B** Similar patterns of survival status and survival time in the training and testing set. **C**, **D** Distribution of m6A-related lncRNA model based on risk score in the high- and low-risk groups plotted in training and testing set. **E**, **F** Kaplan-Meier survival curves of the OS of patients between the high- and low-risk groups in the training and testing set. **G**, **H** Heatmap shows the expression standards of the 6 prognostic lncRNAs of the two sets. **p < 0.01 and ***p < 0.001

In order to verify the accuracy and reliability of prognostic model, we calculated risk scores for every patient in the testing group and training group using the uniform formula. The results displayed the similarly distribution of survival status in the training group and testing group (Fig. 5 A, B), the survival time distribution between high/low risk groups of the testing set was also in accordance with the training set (Fig. 5C, D). Kaplan-Meier survival analysis showed survival probability of the low-risk group was longer than that of the high-risk group in the training set, which was also proved to be ture in the testing set (*p* < 0.001) (Fig. 5E, F). Furthermore, the relative expression of the 6 m6A-related lncRNAs between the low-risk and high-risk groups confirmed that AL391834.1, STARD4-AS1, AC008906.1, and AFF2-IT1 were low-expressed in the high-risk group, the similar pattern was also existed in the testing set, (Fig. 5G, H).

### Validation of the prognostic risk model of m6A-related lncRNAs in clinical features of ML

**Fig. 6:**
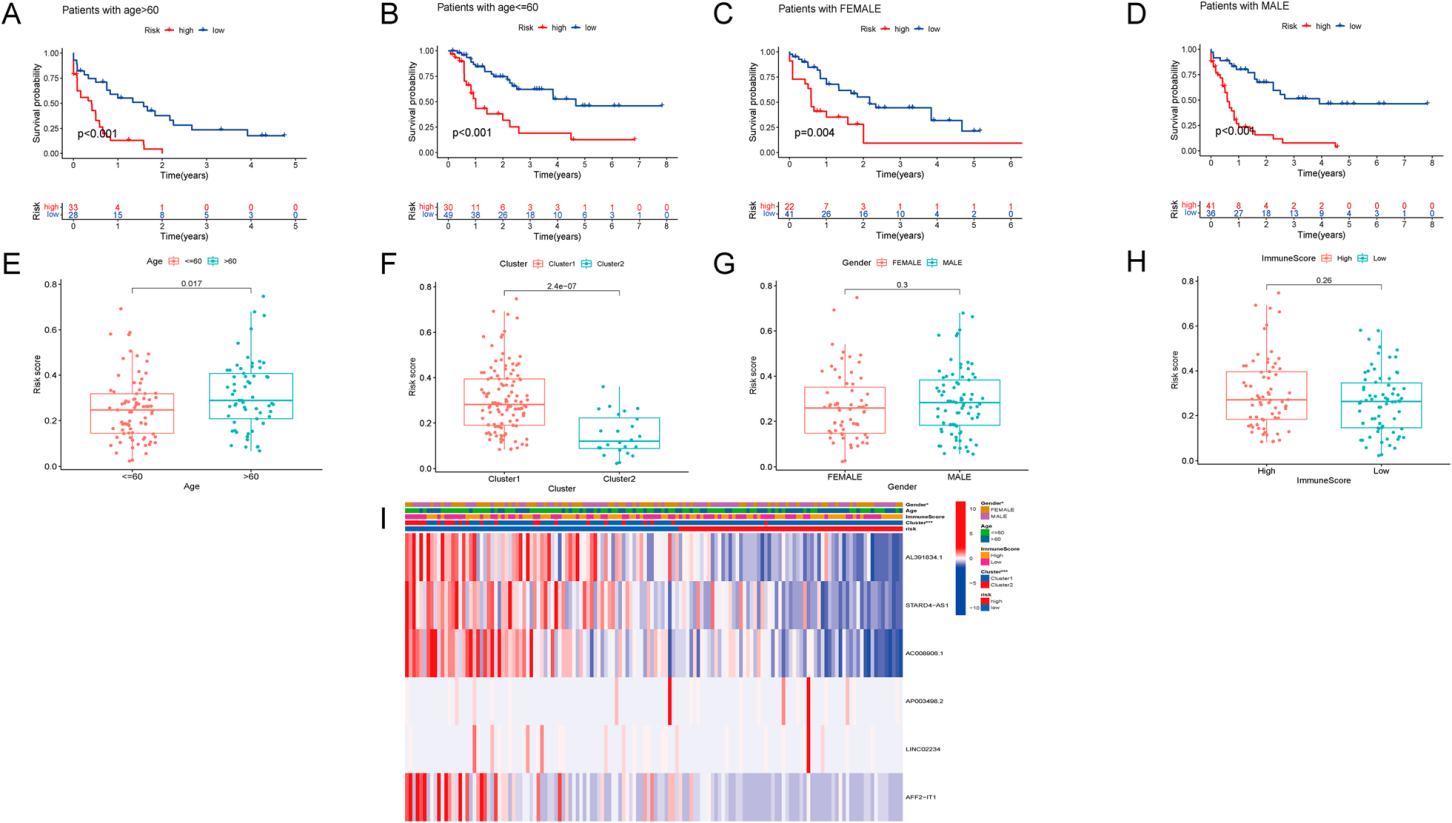
The prognostic risk model was applied to clinical features of ML patients. **A**–**D** Kaplan-Meier curves of OS differences stratified by age, gender between the high- and low-risk groups in the training and testing set. **E**–**H** The risk score was compared between the low- and high-risk groups in the training and testing set. **I** The heatmap of the clinicopathological features (age, gender) and cluster. *p < 0.05 and ***p < 0.001

**Fig. 7:**
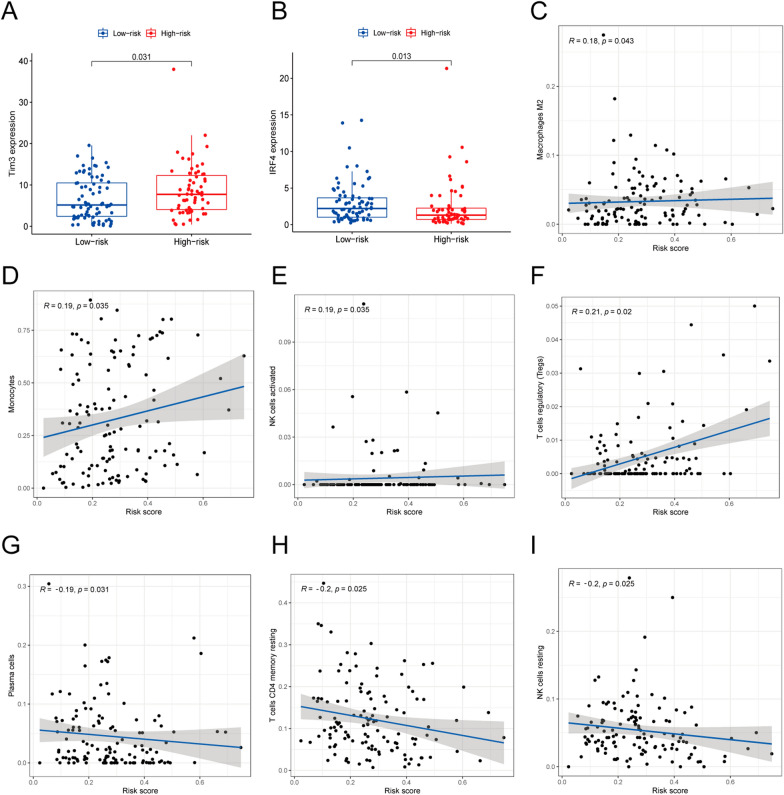
The correlation between immune cell types or immune genes and risk scores. **A**, **B** The relationship between immune genes and risk scores. **C–J** The relationship between immune cell types and risk scores

To evaluate whether our prognostic risk model can be applied to clinical features of ML patients, we divided them into subgroups according to the universal clinicopathological characteristics and compared the difference in OS between the high-risk group and the low-risk group in gender and age. The finding confirmed the OS of the low-risk group was better than the high-risk group, (Fig. 6A–D). Meanwhile, the discrepancies in risk score stratified by gender, age, cluster, and immune score were evaluated in the entire TCGA group. We also revealed that the risk score increased with age, and cluster1 had a higher risk score than cluster2 (Fig. 6E–H). Therefore, the analysis was consistent with the concept that the survival of cluster2 was found to be longer than that of the cluster1 (Fig. 1E). And the heatmap showed that gender and cluster1 were significant factors between the high-risk group and the low-risk group (Fig. 6I). Moreover, we indicated that risk score was also associated with immune genes, the results demonstrated that Tim-3 was up-regulated in the high-risk group, and IRF4 was down-regulated in the low-risk group (Fig. 7A, B).

Finally, we substantiated the correlation between the immune cells and the patient’s survival by analyzing the relationship between immune cells and risk scores. Among them, macrophages M2, monocytes, NK cells activated, and T cells regulatory were positively correlated with the risk score. These results indicated that the higher the content of these cells, the higher the risk score, and the worse the patient’s prognosis (Fig. 7C–G). Conversely, NK cells resting, T cells CD4 memory resting, and plasma cells were negatively correlated with risk scores (Fig. 7H–J).

**Fig. 8:**
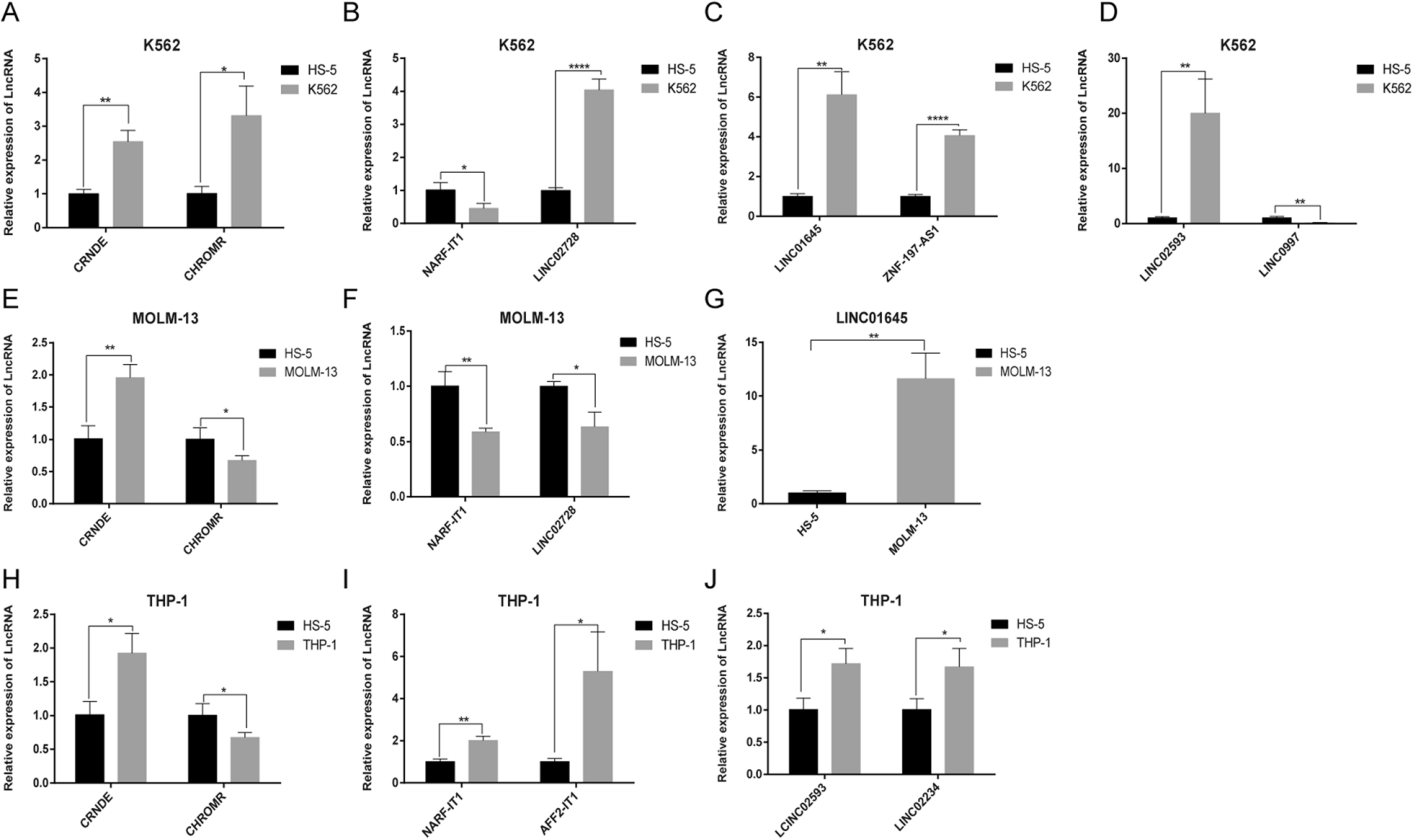
qPCR verification of LncRNA expression in in K562 cells, MOLM-13, and THP-1 cell lines. **A**–**D** The gene expressions of LncRNA were analyzed by real-time PCR in K562 group. **E**–**G** The gene expressions of LncRNA were analyzed by real-time PCR in MOLM-13 group. **H**–**J** The gene expressions of LncRNA were analyzed by real-time PCR in THP1 group. Data were normalized to corresponding GAPDH which expression was used as internal control. *p < 0.05, **p < 0.01, ***p < 0.001, ***p < 0.0001. Difference between two groups were determined by unpaired t-test

Through the above relevant gene prediction by bioinformatics, we then validated the expression levels of LncRNA genes by qPCR in K562 cells, MOLM-13, and THP-1 cell lines. And found that CRNDE was up-regulated, CHROMR and NARF-IT1 had significant differences in the three kinds of cell lines. Most importantly, in myeloid cell lines, LINC02728, and LINC01645 had significant differences, both of which could be predictive biomarkers (Fig. 8A–H).

## Discussion

Although a various of more potent drugs are available, CML and AML disease may still relapse after the drug treatment is stopped. Therefore, increasing researches are involved in further molecular and pathway mechanisms [19]. As a result, it has now been found that lncRNAs can promote tumor growth and metastasis, or play a tumor suppressive role to function as master regulators in various mechanisms and disease processes including ML. Nevertheless, it should be kept in mind that lncRNAs alone may not be enough to drive cell signaling. Similarly, classical signaling molecules may not work alone, or they may not work effectively alone [20]. Although m6A is not as important as signaling molecules themselves to a certain extent, it is clear that m6A appears to be crucial to this regulatory network to make cells, tissues and individuals to better adapt to the environment and survive [21]. Further characterization and connection of these lncRNAs and m6A will provide new ideas for how lncRNAs, m6A, and signaling molecules work together, affecting all aspects of ML.

In this study, we obtained the expression data of 14,086 lncRNAs and 23 m6A genes from the ML TCGA database, and for the first time, an independent model based on m6A-related lncRNAs was constructed to analyze the characterization and link of these lncRNAs and m6A that affecting the survival and risk score of ML. The results showed that 19 m6A genes and 525 LncRNAs were correlated (*p* < 0.001) and demonstrated that 70 m6A-related lncRNAs were related to survival (*p* < 0.01) (Fig. 1B). It would generate more m6A-associated prognostic lncRNAs when *p* < 0.05, which implied that LncRNAs and m6A were extensively regulated and closely linked in ML. The result was consistent with other studies [12]. For instance, m6A modification of lncRNA NEAT1 inhibited cell viability and promoted the apoptosis of CML cells [22].

Meanwhile, the abundance ratio of 22 kinds of immune cells and the scores of stromal cells and immune cells were obtained and calculated between cluster1 and cluster2 in ML. Then, the calculated results demonstrated that immune scores and stromal scores were strongly associated with ML. These terms: T cells CD4 memory resting, T cells follicular helper, Mast cells resting and Mast cells activated cover a small proportion; T cells CD4 memory activated and Monocytes accounts for a larger proportion in cluster1. It was further speculated that the content of stromal cells and immune cells would correlate with the survival of patients with ML. Likewise, stromal cells and immune cells content also had a correlation with the survival of patients with cervical cancer, colorectal cancer, and epithelial ovarian cancer [23–25]. Subsequently, this important finding further emphasizes immune genes (PD-L1, IRF4, IRX3, LAG-3, Tim-3 and NR4A1) were also associated with ML.

We then used LASSO-penalized Cox analysis to construct a prognosis model related to m6A-LncRNAs of ML patients, and we confirmed that the m6A-related lncRNAs model, whether in the training or testing group, was an autocephalous risk element of OS using univariate and multivariate Cox regression analysis. And the results showed that the low-risk group based on the intermediate risk score had better prognosis. Then, prognostic risk model of m6A-related lncRNAs was validated to have excellent consistency in clinical features of ML. Simultaneously, we indicated that risk score was also associated with immune genes and cells, that Tim-3 was up-regulated in the high-risk group, and IRF4 was down-regulated in the low-risk group. Previous studies also proposed that TIM-3/Gal-9 could activate both the NF-κB and the β-catenin signaling and promote a range of leukemic progression in AML [26–28]. Besides, IRF4 could have regulated myeloid and lymphoid hematopoietic differentiation, and absence of it would exacerbate the development of myeloid leukemia [29, 30]. These research results were consistent with our data in the validation of m6A-related lncRNA model. In addition, the heatmap illustrated AL391834.1, STARD4-AS1, AC008906.1, and AFF2-IT1 were low-expressed in the high-risk group. Among them, all lncRNAs were discovered for the first time.

By further verifying the expression of m6A-related lncRNAs in cell lines, we found that CRNDE was up-regulated, CHROMR and NARF-IT1 had significant differences in the three cell lines. In myeloid cell lines, LINC02728, and LINC01645 had significant differences, both which could be predictive biomarkers. Some reports had proved lncRNA CRNDE could promote AML cell proliferation [31, 32]; and lncRNA CRNDE also promoted colorectal cancer cell proliferation, chemoresistance, and it attenuated chemoresistance in gastric cancer [33, 34]. These results were sufficient to prove the accuracy and authenticity of the m6A-related lncRNAs model.

To summarize, our research provided clues for the prognosis prediction of ML patients, and clarified the accuracy and authenticity of the m6A-related lncRNAs model. In addition, we elucidated more on mechanisms between m6A-regulated lncRNAs and ML. Most importantly, further exploration of interaction between LncRNAs and genes needs more basic experiments and clinical samples to be included in our follow-up experiments.

## Supplementary Information

**Additional file 1: Table S1.** The specific data information and cluster grouping of clinical samples.

**Additional file 2: Table S2.** Sequence of specific PCR primers of different target genes.

**Additional file 3: Table S3.** The specific raw data information of clinical samples in the training group and testing group.

## Data Availability

The datasets generated and/or analyzed during the current study were TCGA (https://portal.gdc.cancer.gov/). The datasets used and analyzed during the current study are available from the corresponding author on reasonable request.

## Abbreviations

CML: Chronic myeloid leukemia
AML: Acute myeloid leukemia
m6A: N6-methyladenosine
DEGs: Differentially Expressed Genes
qRT-PCR: Quantitative real-time
