# Supplementary material for: The prognosis biomarkers based on m6A-related lncRNAs for myeloid leukemia patients
Source: Cancer Cell Int. 2022 Jan 7;22:10. doi: 10.1186/s12935-021-02428-3 (PMC8739709; doi:10.1186/s12935-021-02428-3)
Supplement: Supplementary file 2 — Additional file 2: Table S2. Sequence of specific PCR primers of different target genes. [file 12935_2021_2428_MOESM2_ESM.doc]

Supplementary

Table S1. Sequence of specific PCR primers of different target genes

| *Genes* | Sequence(5′→3′) |
| --- | --- |
| CRNDE | Forward：TCAGCCGTTGGTCTTTGAAAT |
|  | Reverse：TCTTCTGCGTGACAACTGAGG |
| *CHROMR* | Forward：TCTTTAGGGGTTCCCGGACT |
|  | Reverse：GTTCTTCAGCCACCGCTACT |
| *NARF-IT1* | Forward：CACAGGCCCCTGACTTGAAA |
|  | Reverse：CTGAGAGTGAAGGATGGCCG |
| *LINC02728* | Forward：TGTGGTGGTTGCCGAAGAA |
|  | Reverse：TGCCTCATAACGCTGTCTGT |
| *AFF2-IT1* | Forward：TTAGCACTGATTGGGTCTCAGTT |
|  | Reverse：CCGTCATGTTCTCTGTGTAGC |
| *LINC01645* | Forward：ACATCTGGGAACTACCGCCA |
|  | Reverse：TCATCACCACTGCACTCTTCC |
| *ZNF197-AS1* | Forward：TGGGGAATCCTCCTATGTCCT |
|  | Reverse：GCCATTGAGGTATGCACTCTG |
| *LINC02593* | Forward：CTTGTTCTGAGGCCCCTAGC |
|  | Reverse：TGTTACGCACGTTCTGGTCT |
| *LINC02234* | Forward：TGCCGTGCTATCTTTCCTACC |
|  | Reverse：CACAAGCACGGTTTGGTGAG |
| *LINC00997* | Forward：ATTCCCTAGCATTGCAGCCTC |
|  | Reverse：GTGCTCAGCGATTTCTACCC |
| *GAPDH* | Forward：CTGGGCTACACTGAGCACC |
|  | Reverse：AAGTGGTCGTTGAGGGCAATG |
